# Impacts of baseline biomarkers on cognitive trajectories in subjective cognitive decline: the CoSCo prospective cohort study

**DOI:** 10.1186/s13195-023-01273-y

**Published:** 2023-08-07

**Authors:** Yun Jeong Hong, SeongHee Ho, Jee Hyang Jeong, Kee Hyung Park, SangYun Kim, Min Jeong Wang, Seong Hye Choi, Dong Won Yang, CoSCo study group

**Affiliations:** 1 Department of Neurology, Uijeongbu St. Mary’s Hospital, The Catholic University of Korea, Seoul, South Korea; 2 Department of Neurology, Seoul St. Mary’s Hospital, The Catholic University of Korea, Seoul, South Korea; 3 Department of Neurology, Ewha Womans University Seoul Hospital, Ewha Womans University College of Medicine, Seoul, South Korea; 4 Department of Neurology, Gachon University Gil Hospital, Incheon, South Korea; 5 Department of Neurology, Seoul National University College of Medicine, Seoul National University Bundang Hospital, Seongnam, South Korea; 6 Department of Neurology, Roa Neurology Clinic, Seongnam, South Korea; 7 Department of Neurology, Inha University School of Medicine, Incheon, South Korea

**Keywords:** Subjective cognitive decline, Alzheimer’s disease, Biomarker, Cohort study, Cognitive decline

## Abstract

**Background:**

Subjective cognitive decline (SCD) is a risk factor for Alzheimer’s disease (AD); however, the rates of cognitive decline are variable according to underlying pathologies and biomarker status. We conducted an observational study and aimed to investigate baseline characteristics and biomarkers related with cognitive declines in SCD. Our study also assessed whether SCD participants showed different cognitive and biomarker trajectories according to baseline amyloid deposition.

**Methods:**

This study is a part of a longitudinal cohort study conducted in multi-centers in South Korea between 2018 and 2021. Individuals (≥ 60 years old) with persistent cognitive complaint despite of normal cognitive functions were eligible for the study. All participants underwent neuropsychological tests, florbetaben PET scans, plasma amyloid markers, and brain MRI scans. Annual follow-up evaluations included neuropsychological tests and assessments for clinical progressions. Regional brain volumetry and amyloid burden represented by PET-based standardized uptake value ratio (SUVR) were measured. We compared cognitive and brain atrophic changes over 24 months between amyloid positive-SCD (Aβ + SCD) and amyloid negative-SCD (Aβ-SCD) groups. Baseline factors associated with cognitive outcomes were investigated.

**Results:**

A total of 120 participants with SCD were enrolled and 107 completed follow-up evaluations. Aβ + SCD participants (*n* = 20, 18.5%) were older and more frequently APOE4 carriers compared with Aβ-SCD participants (*n* = 87). Baseline cognitive scores were not different between the two groups, except the Seoul Verbal Learning Test (SVLT) scores showing lower scores in the Aβ + SCD group. After 24 months, plasma amyloid markers were higher, and regional volumes (entorhinal, hippocampal, and pallidum) were smaller in the Aβ + SCD participants compared with Aβ-SCD participants adjusted by age, sex, and baseline volumes. SVLT delayed recall and controlled oral word association test (COWAT) scores indicated more declines in Aβ + SCD participants. Baseline left entorhinal volumes were related to verbal memory decline, while baseline frontal volumes and global SUVR values were related to frontal functional decline.

**Conclusion:**

Aβ + SCD participants showed more cognitive decline and medial temporal atrophic changes during 24 months. Baseline neurodegeneration and amyloid burden were related with future cognitive trajectories in SCD.

**Trial registration:**

This study was registered at CRIS (KCT0003397).

**Supplementary Information:**

The online version contains supplementary material available at 10.1186/s13195-023-01273-y.

## Introduction

Subjective cognitive decline (SCD) is defined as self-reported persistent cognitive decline without objective cognitive impairment. SCD is a risk group for dementia and Alzheimer’s disease (AD) [1, 2]; however, the cognitive trajectories are variable due to the heterogeneity of underlying pathologies [3, 4]. Hence, the identification of biomarker status and baseline factors related with underlying pathologies is clinically important to predict future clinical progression.

In previous studies, multiple risk factors for AD dementia have been reported in SCD including old age, apolipoprotein epsilon 4 (APOE4) allele, concern about cognitive declines, informant’s report of the cognitive decline, existence of neurodegenerations, and amyloid depositions at baseline [4–8]. In amyloid-positive SCD (Aβ + SCD) subjects, the rates of clinical progression to mild cognitive impairment (MCI) or dementia increases up to 40–62% over approximately 5 years [3], which suggests that these subjects are the later stages of preclinical AD. We planned a prospective cohort study to observe clinical progression and assess baseline predictors related with clinical progression in elderly participants with SCD. In our previous study [4], Aβ + SCD participants showed faster verbal memory decline compared with Aβ-SCD participants over 24 months. We hypothesized that baseline amyloid burden might be associated with future cognitive decline in SCD [4]. However, our previous study had several limitations, including a small sample size, sampling bias in a single center, and lack of biomarker information except the baseline amyloid positron emission tomography (PET) finding. A further prospective cohort study with a larger sample size of SCD participants from multiple centers and sufficient biomarker evaluations was needed to clarify the previous results and hypothesis.

Here, we planned a longitudinal observational study and aimed to assess baseline characteristics and biomarkers related with clinical progression in participants with SCD. We also assessed whether SCD participants showed different cognitive and biomarker trajectories according to baseline amyloid depositions.

## Methods

### Study design

This study is part of a prospective longitudinal cohort study named “COhort study to identify predictors for the clinical progression to mild cognitive impairment or dementia from Subjective COgnitive decline (CoSCo).” Detailed study design is described in a previous report [9]. In brief, the CoSCo study was conducted in six centers in South Korea and enrolled SCD participants between November 2018 and December 2021. Florbetaben PET scans were performed in all participants to determine amyloid burden at baseline. Demographic characteristics, brain magnetic resonance imaging (MRI), detailed neuropsychological tests, physical and neurological examinations, biological data using wearable device monitoring, questionnaires assessing the subjective complaints, and plasma amyloid beta values were evaluated at baseline and follow-up visits. All participants underwent annual follow-up evaluations to assess cognitive changes/clinical progression until the endpoint (24 months). Annual follow-up evaluations included detailed neuropsychological tests, neurological and physical examinations, and questionnaires assessing the subjective complaints. Plasma amyloid beta values and brain MRIs were obtained at baseline and endpoint.

### Participants

Individuals who were diagnosed with SCD were eligible for the study. Inclusion criteria were as follows: (1) age 60 years old or older, (2) complaint of persistent cognitive decline, (3) normal performance in detailed neuropsychological tests in the Seoul Neuropsychological Screening Battery (SNSB) [10], (4) performance range between 7 to 50th percentile (a standardized score adjusted by age, sex, and education) of the verbal memory delayed recall function test named Seoul Verbal Learning Test (SVLT) [10], (5) clinical dementia rating (CDR) [11] score of 0; (6) literate; and (7) agreed to participate in the study and was able to visit the hospital for annual evaluations. The exclusion criteria were the following: (1) any unstable or severe medical condition (e.g., severe hepatic or renal disease, unstable cardiovascular disease, severe asthma, active gastric ulcer, cancer); (2) neurological disorders such as Parkinson’s disease, Huntington’s disease, or normal pressure hydrocephalus; (3) major psychiatric disorders such as uncontrolled depression, schizophrenia, alcoholism, or drug dependency; (4) mild cognitive impairment (MCI) or dementia; (5) abnormal blood laboratory findings such as abnormal thyroid function, low vitamin B_12_ or low folate, or positive syphilis serology; and (6) brain lesions known to cause cognitive impairment (tumor, stroke, or subdural hematoma). We included SCD participants with relatively lower verbal memory scores (below the 50th percentile adjusted by age, sex, and education) considering that memory decline is a major presenting symptom of typical AD^5^ and lower SVLT score is related with higher progression rate in SCD participants [6]. We divided participants into 2 groups: group 1, Aβ-positive SCD (Aβ + SCD) (global standardized uptake value ratio (SUVR) ≥ 1.391), and group 2, Aβ-negative SCD (Aβ-SCD) (global SUVR < 1.391).

### Neuroimaging

All participants underwent brain MRI and florbetaben PET scans at baseline. Brain MRI scans were performed again at the endpoint visit to evaluate neurodegenerative changes. Regional volumetry and assessments for small vessel disease findings were performed using brain MRIs. Visual ratings for amyloidosis and measurements of quantitative amyloid burden represented by SUVR were performed using florbetaben PET scans.

### MRIs and regional volumetry

Brain MRI scans were performed using a 3.0-Tesla scanner (GE Medical Systems, Milwaukee, WI, USA), including fluid attenuated inversion recovery (FLAIR), susceptibility weighted images, and three-dimensional (3D) T1-weighted images (WI). The white matter hyperintensities (WMHs) were rated using a visual rating scale of axial FLAIR images. In brief, periventricular WMHs and deep WMHs were evaluated separately and rated as minimal (grade 1), moderate (grade 2), or severe (grade 3) [12]. Lacunes were defined as small lesions (3–15 mm in diameter), hyperintense on T2-WI, and hypointense on T1-WI, with a perilesional halo on FLAIR [13]. Cerebral cortical microbleeds were defined as round and low-signal lesions (less than 10 mm in diameter) in lobar areas on susceptibility weighted images [13]. Hippocampal atrophy was rated on coronal T1-WI using Scheltens’ visual rating scale [14]. The mean of the left and right hippocampal atrophy scores was used. The degree of hippocampal atrophy, number of lacunes, number of microbleeds, and degree of WMH were measured by a neurologist (S.H. Ho) blinded to the data. The MRI processing and volumetric analysis were performed using AQUA 2.0 program (Neurophet, South Korea). The details of the MRI segmentation and data analysis were described elsewhere [15]. A normative dataset was obtained using the East-Asian dataset described in a previous study [16], and the adjusted volume (*z* score) corrected with total intracranial volume, age, and sex is measured.

### Florbetaben PET

Florbetaben (18F) PET scans were acquired following the standardized protocol [17, 18]. Using PET scans, a whole brain visual interpretation was performed by a trained specialist in nuclear medicine who was blinded to the diagnosis. The brain amyloid plaque load (BAPL) score, a rating scale of florbetaben PET [17, 18], was used to categorize amyloid-positive and amyloid-negative participants. BAPL scores of 2 or 3 indicated a positive finding for amyloidosis according to the reference [17, 18].

Quantitative neuroimaging analyses were performed using PET scans and MRI 3D-T1 images. First, amyloid depositions were assessed using MATLAB version 2013a and SPM8 (http://www.fil.ion.ucl.ac.uk/spm/software/spm8). Individual 3D T1-WI scans were estimated and co-registered into corresponding PET images. A volume-based template, incorporating 90 regions-of-interest (ROI), named automatic anatomical labeling (AAL), was aligned to individual T1-WI [19]. The voxels of florbetaben PET images were scaled using the mean uptake value in the cerebellar cortex to calculate the SUVR, and partial volume corrections (PVC) were performed. For PVC, the voxels located in gray matter with a probability less than 20% were discarded in each PET image. We selected 28 AD-specific cortical ROIs from the AAL atlas according to the previous methods [20], and the mean SUVR values were calculated as a global SUVR.

### Baseline and follow-up cognitive tests

All participants were diagnosed with SCD using the formal neuropsychological test battery SNSB [10], including the Korean version of the Mini-Mental State Examination (K-MMSE) [21], CDR, Korean version of the instrumental activities of daily living (K-IADL) scale [22], attention (digit span test), Boston naming test, tests for comprehension/repetition/fluency, visuospatial function using Rey Complex Figure Test (RCFT), verbal and visual memory function tests (SVLT and RCFT), and frontal executive function tests using contrasting program, go-no-go, Controlled Oral Word Association Test, Stroop test, and trail making test (TMT) [10]. The percentile scores, standardized scores adjusted by age, sex, and education, are based on a large nationwide Korean sample (1100 people), making it possible to perform comparisons with the population averages. Scores ≥ 16th percentile, which were compared to –1 standard deviation (SD) of the norm, were defined as normal. Severity of the cognitive complaints was assessed using Korean-everyday cognition (ECOG); higher total score indicates more cognitive complaints [23].

The annual follow-up evaluations (a visit window up to 3 months was allowed) included SNSB, neurological and physical examinations, and physician’s history taking to assess clinical progression to MCI or dementia. The cognitive tests were administered by trained neuropsychologists. Progression to MCI/dementia was evaluated based on follow-up neuropsychological tests and history takings in an outpatient clinic. Participants with CDR score ≥ 0.5 or SVLT delayed recall scores < 7th percentile scores or any cognitive function scores (except the SVLT delayed recall) < 16th percentile in the SNSB were considered to have progressed to MCI or dementia.

### Plasma amyloid beta values

Plasma amyloid beta values were measured using the Multimer Detection System-oligomeric Aß (MDS-OAß) method [24]. In brief, the inBloodTM™ OAß test (People Bio Inc., Gyeonggi-do, Republic of Korea) was used to quantify MDS-OAß values in EDTA vacutainer tubes. Higher values indicate more amyloid oligomeric tendencies with vigorous amyloidosis. According to a previous study, a plasma amyloid beta value ≥ 1.00 was considered positive for a plasma amyloid marker [24].

### Statistical analysis

Using receiver operating characteristic (ROC) curves, the cut-off values of global SUVR discriminating PET-positivity and PET-negativity on the visual rating scale with the highest Youden’s index of excellent area under curve (AUC) were determined in the study participants.

We compared cognitive and neurodegenerative changes over 24 months between the two groups. Independent *t*-test or nonparametric Mann–Whitney *U* test (based on normal distribution patterns) was used for the comparison of continuous variables between Aβ + SCD and Aβ-SCD subjects. Chi-square tests were used to compare categorical variables. Repetitive measure ANOVA was used to compare cognitive changes during the study periods between the two groups. To assess relevant baseline factors associated with cognitive changes, multivariable linear regression analysis was performed. All statistical analyses were performed using SPSS (version 18.0; SPSS Inc., Chicago, IL, USA). *p*-values < 0.05 were considered to indicate statistically significant differences.

## Results

### Baseline characteristics

**Fig. 1:**
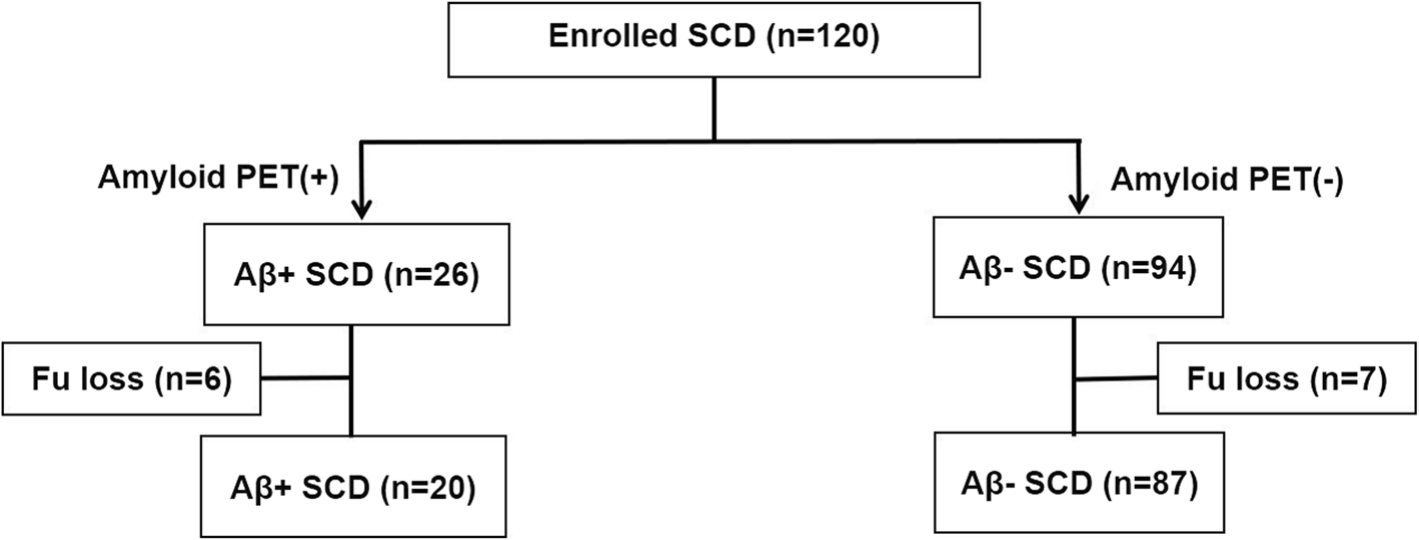
Flowchart of participant selection

A total of 120 elderly participants with SCD were enrolled and 107 participants completed the study (Fig. 1). Nine participants (8.4%) progressed to MCI/dementia during the study period. There were no differences in baseline characteristics between the participants who completed the study and the subjects who dropped out, except educational levels (Supplementary Table 1). Global SUVR values and ECOG scores were higher in the participants who completed the study compared with those who dropped out (Supplementary Table 1).

The optimal cut-off value of global SUVR was determined as 1.391 with an excellent AUC (0.968) and Youden index (0.748). The cut-off value was used to divide participants with SCD into Aβ + SCD and Aβ-SCD groups. Aβ + SCD participants (*n* = 20) were older, had higher educational levels, and were more frequently APOE4 carriers (Table 1) compared with Aβ-SCD participants (*n* = 87). Waist circumference, body fat, and visceral fat were lower, but cardiovascular risk scores were higher in the Aβ + SCD group compared with the Aβ-SCD group. Baseline neuropsychological tests scores were not different between the two groups, except the SVLT delayed recall scores, which were lower in the Aβ + SCD group (Table 1).

**Table 1:** Baseline characteristics between the groups

Variables	Aβ-SCD (*n* = 87)	Aβ + SCD (*n* = 20)	*p*
Female (*n*, %)	52 (59.8%)	7 (35.0%)	0.079
Age, years	69.77 ± 5.89	74.95 ± 5.72	0.001
Education, years	11.2 ± 4.1	13.2 ± 3.4	0.040
APOE4 carrier (*n*, %)	11 (12.6%)	10 (50.0%)	0.001
Waist circum, cm	87.1 ± 9.1	81.6 ± 9.2	0.015
Body fat (%)	29.8 ± 7.7	25.7 ± 8.7	0.037
Body muscle (%)	24.7 ± 6.7	23.7 ± 4.8	0.551
Visceral fat (%)	9.6 ± 3.6	6.5 ± 3.0	0.001
Global SUVR	1.198 ± 0.113	1.642 ± 0.202	< 0.001
MDS_Plasma amyloid βeta	0.851 ± 0.199	0.961 ± 0.122	0.030
Deep WMH (mild/moderate/severe)	64/16/7	16/4/0	0.423
Periventricle WMH (mild/moderate/severe)	60/17/10	13/3/4	0.723
Lacunes (*n*)	0.09 ± 0.328	0.05 ± 0.224	0.589
Microbleed (*n*)	0.06 ± 0.234	0.15 ± 0.366	0.291
Framingham cardiovascular risk score	8.3 ± 6.9	12.7 ± 8.8	0.017
Baseline K-MMSE score	27.4 ± 1.9	26.9 ± 2.1	0.402
DST-Forward_percentile	63.1 ± 29.4	60.2 ± 32.3	0.691
K-BNT_percentile	60.6 ± 25.0	60.6 ± 32.9	0.995
RCFT Copy_percentile	58.5 ± 21.6	58.5 ± 22.2	0.992
SVLT delayed recall_percentile	30.0 ± 14.3	20.1 ± 12.5	0.005
RCFT delayed recall_percentile	48.5 ± 24.5	45.1 ± 25.3	0.572
Digit symbol constitution_score	63.4 ± 24.8	50.3 ± 34.0	0.051
COWAT phonemic_percentile	53.3 ± 28.0	55.2 ± 31.0	0.788
K-TMT-B_percentile	61.6 ± 22.2	61.8 ± 20.5	0.983
K-stroop color reading_percentile	56.0 ± 24.6	49.6 ± 29.8	0.317
K-ECOG memory total	17.7 ± 5.7	18.1 ± 4.9	0.819
K-ECOG total	72.1 ± 22.1	72.1 ± 20.5	0.999

*Abbreviations: SCD* Subjective cognitive decline, *APOE4* Apolipoprotein epsilon 4, *SUVR* Standardized uptake value ratio, *MDS* Multimer detection system, *WMH* White matter hyperintensities, *K-MMSE* Korean version of Mini-Mental State Examination, *DST* Digit span test, *BNT* Boston naming test, *RCFT* Rey complex figure test, *SVLT* Seoul verbal learning test, *COWAT* Controlled Oral Word Association Test, *TMT* trail making test, *ECOG* Everyday cognition

### Baseline regional amyloid burdens and neurodegeneration

In regard to neurodegeneration represented by regional volumes, Aβ + SCD participants showed smaller left hippocampus and pallidum and larger frontal lobes compared with Aβ-SCD participants adjusted by the individual’s intracranial volume, age, and sex (Supplementary Table 2). Other regional volumes were similar between the two groups (Supplementary Table 2).

In regard to regional amyloid burdens represented by SUVRs, Aβ + SCD participants showed significantly higher amyloid depositions (except the hippocampus, caudate, and thalamus) and plasma amyloid beta values (Table 1, Supplementary Table 2).

### Cognitive and biomarker trajectories according to amyloid status

**Fig. 2:**
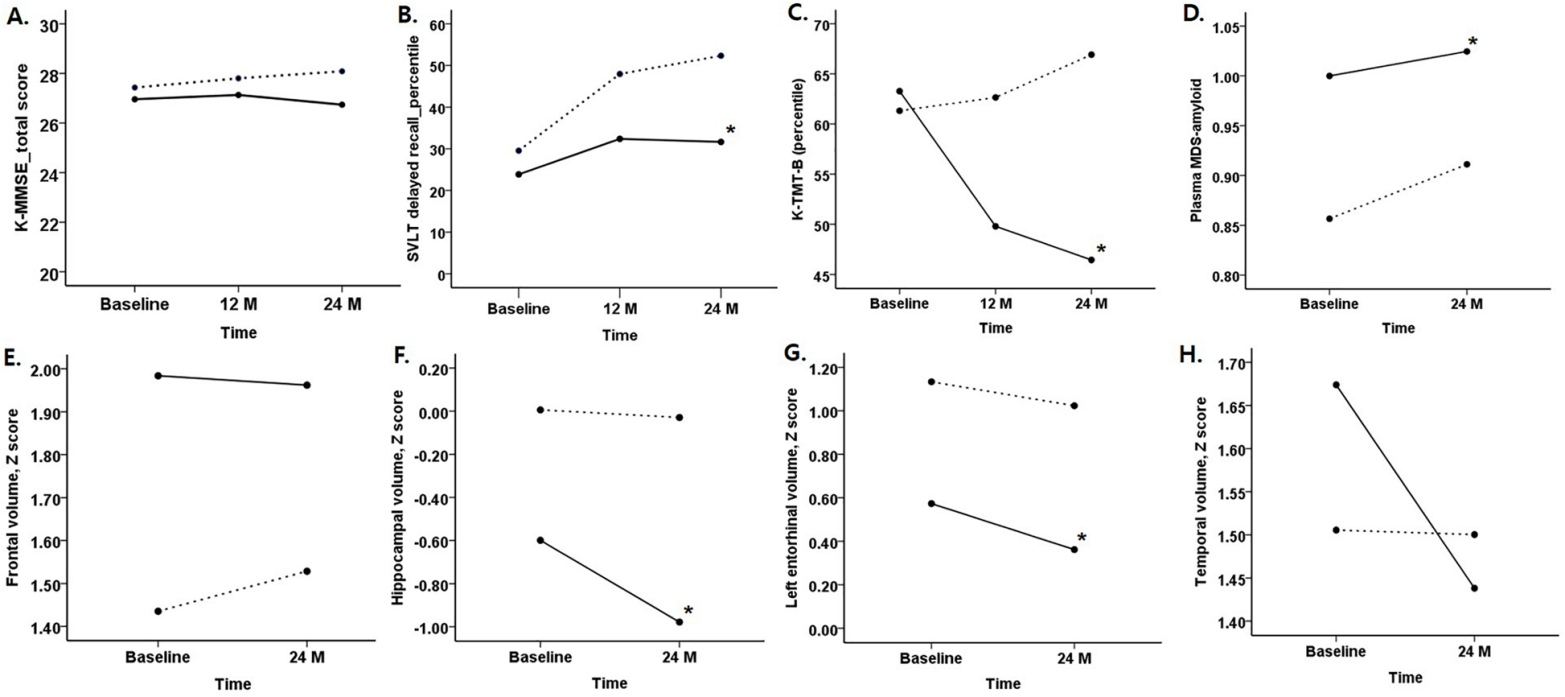
Cognitive and biomarker changes during 24 months between amyloid-positive SCDs and amyloid-negative SCDs. Dotted line indicates Aβ-SCD and solid line indicates Aβ + SCD * Statistically significant (*p* < 0.05)

Cognitive changes according to baseline amyloid status are shown in Table 2. After 12 months, SVLT delayed recall scores and TMT-B scores declined more in the Aβ + SCD participants compared with those in the Aβ-SCD participants adjusted by age, sex, and education (Table 2). At the endpoint (after 24 months), the two cognitive domain scores (SVLT delayed recall and TMT-B scores) showed more prominent decline in the Aβ + SCD participants compared with those in the Aβ-SCD participants adjusted by age, sex, and education (Table 2, Fig. 2B, C). K-MMSE scores were not different according to amyloid status (Fig. 2A).

**Table 2:** Cognitive changes during 24 months

Variables	Aβ-SCD (*n* = 87)	Aβ + SCD (*n* = 20)	*p*
***At 12 months***			
DST-Forward_percentile	63.475 ± 31.659	65.739 ± 30.784	0.777
K-BNT_percentile	59.491 ± 26.719	64.559 ± 32.940	0.475
RCFT Copy_percentile	51.246 ± 25.419	60.095 ± 18.986	0.156
SVLT delayed recall_percentile	47.467 ± 26.922	30.507 ± 25.009	0.013
RCFT delayed recall_percentile	52.414 ± 30.292	52.541 ± 28.228	0.987
Digit symbol constitution_score	65.205 ± 26.403	64.417 ± 30.637	0.909
COWAT phonemic_percentile	54.044 ± 27.552	60.327 ± 32.563	0.386
K-TMT-B_percentile	62.677 ± 23.257	49.680 ± 28.259	0.036
K-stroop color reading_percentile	60.766 ± 25.958	53.286 ± 31.261	0.276
K-MMSE score	27.765 ± 1.968	27.158 ± 2.292	0.241
***At 24 months***			
DST-Forward_percentile	68.293 ± 28.429	62.637 ± 31.599	0.434
K-BNT_percentile	65.726 ± 27.441	56.719 ± 37.362	0.221
RCFT Copy_percentile	52.100 ± 25.450	55.395 ± 23.359	0.597
SVLT delayed recall_percentile	51.010 ± 25.863	31.738 ± 23.934	0.003
RCFT delayed recall_percentile	58.345 ± 29.150	52.692 ± 28.664	0.435
Digit symbol constitution_score	68.433 ± 25.238	61.783 ± 34.289	0.325
COWAT phonemic_percentile	55.173 ± 29.411	59.796 ± 30.297	0.530
K-TMT-B_percentile	67.452 ± 21.089	45.566 ± 26.767	< 0.001
K-stroop color reading_percentile	60.524 ± 28.679	53.032 ± 27.412	0.291
K-MMSE score	28.047 ± 1.775	26.700 ± 2.830	0.054

*Abbreviations*: *SCD*, subjective cognitive decline; *K-MMSE*, Korean version of Mini-Mental State Examination; *DST*, digit span test; *BNT*, Boston naming test; *RCFT*, Rey complex figure test; *SVLT*, Seoul verbal learning test; *COWAT*, Controlled Oral Word Association Test; *TMT*, trail making test

After 24 months, entorhinal, hippocampal, and pallidum volumes were significantly smaller (*p* < 0.05) in the Aβ + SCD participants compared with Aβ-SCD participants adjusted by age, sex, and baseline volumes (Supplementary Table 3, Fig. 2F, G). Plasma MDS-amyloid beta values were significantly higher in the Aβ + SCD participants compared with Aβ-SCD participants after adjustment for baseline MDS-amyloid beta values (Supplementary Table 2, Fig. 2D).

When participants were further divided according to plasma amyloid beta values (plasma Aβ-SCD (*n* = 76) and plasma Aβ + SCD (*n* = 29)), the 24 months cognitive trajectories were not different between the two groups (*p* > 0.05, data not shown).

### Cognitive and biomarker trajectories according to baseline preclinical stages

**Fig. 3:**
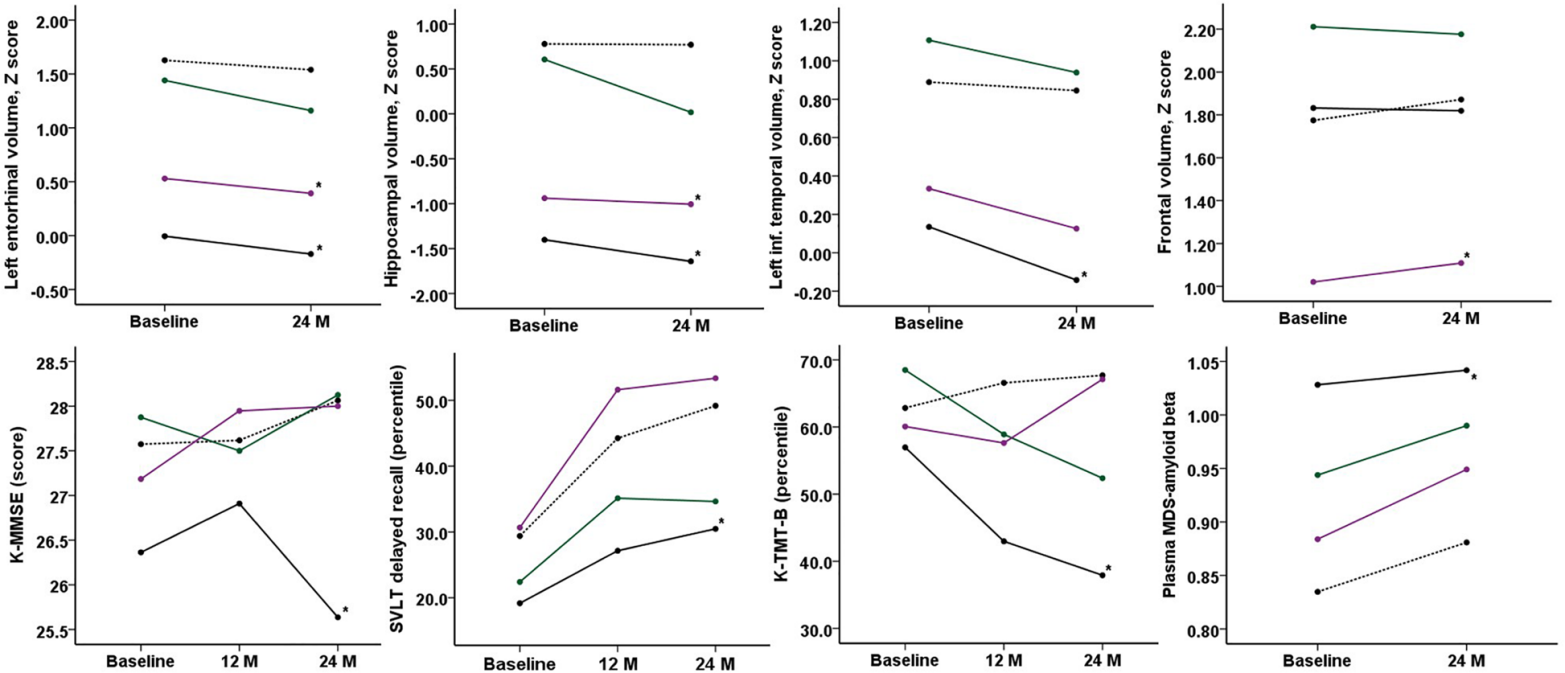
Cognitive and biomarker changes during 24 months among the preclinical groups. Black dotted line: stage 0, green line: stage 1, black line: stage 2, purple line: SNAP * Statistically significant difference compared with the group 0

We additionally divided the participants into 4 groups according to baseline preclinical stage [25]: Aβ-neurodegeneration- (stage 0, *n* = 49), Aβ + neurodegeneration- (stage 1, *n* = 8), Aβ + neurodegeneration + (stage 2, *n* = 12), and Aβ- neurodegeneration + (SNAP, *n* = 38). Participants with regional volumetric *Z* scores below − 1.0 adjusted by age, sex, and intracranial volumes in any areas were considered to have neurodegenerations. Baseline age, APOE4 allele status, RCFT delayed recall score, and digit symbol constitution scores were different among the preclinical stage groups (Supplementary Table 3). Participants with SCD of stage 2 were the oldest and participants with SCD of stage 0 were the youngest at baseline (Supplementary Table 3). After 24 months, K-BNT scores were different among the groups, with the lowest score in the stage 2 group and the highest score in the stage 0 group after adjustment for age, sex, and education (Supplementary Table 4). In comparing cognitive and biomarker trajectories among the 4 preclinical stages, changes in K-MMSE score, SVLT delayed recall scores, K-TMT-B scores, medial temporal volumes (left entorhinal and hippocampal volumes), left inferior temporal volumes, frontal volumes, and plasma MDS-amyloid beta values were different according to baseline biomarker groups (Fig. 3). Participants with SCD of stage 2 showed lower scores in the K-MMSE, K-TMT-B, and the SVLT delayed recall tests compared with participants with SCD of stage 0 (Fig. 3E, F, G). Participants with SCD of stage 2 exhibited more atrophic changes in the medial and inferior temporal lobes (Fig. 3A, B, C), while participants with SCD of SNAP exhibited the smallest frontal lobes compared with the other groups (Fig. 3D).

### Relationships between baseline factors and cognitive/biomarker trajectories

In multiple regression analysis, baseline SVLT delayed recall scores and entorhinal volumes were factors related with verbal delayed recall outcomes (Supplementary Table 5–1). Baseline K-TMT-B scores, baseline frontal volumes, and baseline global SUVR values were factors related with frontal executive functional outcomes represented by K-TMT-B scores (Supplementary Table 5–2). For MMSE score outcomes, baseline MMSE scores, APOE4 allele existence, and baseline inferior temporal volumes were relevant factors (Supplementary Table 5–3).

## Discussion

In this study, we performed a longitudinal observational study to assess cognitive and biomarker changes in Korean participants with SCD over the course of 24 months. We investigated whether SCD participants showed different cognitive and biomarker trajectories according to baseline biomarker findings.

Our study revealed three major findings. First, Aβ + SCD participants showed different cognitive and biomarker trajectories compared with Aβ-SCD participants. Verbal memory delayed recall function represented by SVLT delayed recall scores and frontal executive function represented by TMT-B scores showed prominent declines in Aβ + SCD participants compared with those in Aβ-SCD participants. The differences between the two groups were more prominent at 24 months than at 12 months. Regarding biomarker changes, medial temporal and pallidum volumes were significantly smaller after 24 months in the Aβ + SCD participants compared with Aβ-SCD participants adjusted by age, sex, and baseline volumes. SCD with Alzheimer’s pathologic changes showed greater cognitive decline and neurodegenerative changes despite of the relatively short follow-up durations. Second, cognitive and biomarker trajectories were different according to baseline neurodegeneration. Participants with Aβ + SCD with neurodegeneration in any areas (stage 2) showed rapid cognitive decline compared with those with Aβ + SCDs without neurodegeneration (stage 1). However, neurodegeneration without amyloidosis, the SNAP group, showed similar cognitive and biomarker changes with all negative participants (stage 0), suggesting that neurodegeneration is proxy of cognitive decline and amyloidosis might be the leading key step of future rapid cognitive decline. Neurodegeneration combined with sufficient amyloid burden above the threshold level would show cognitive progression in SCD. Third, baseline biomarkers including amyloid burden and regional volumes are prominently related with future cognitive outcomes, while other clinical characteristics did not show significant relationships with cognitive outcomes in our study. According to the results, baseline regional brain volumes and cognitive performance are related to future cognitive decline; moreover, baseline amyloid depositions are additional factor related with future frontal executive functional decline. Intriguingly, TMT-B scores, a frontal functional outcome, were negatively related with baseline global SUVR values independently of frontal volumes. Our results can be explained by the assumption that TMT-B scores not only represent frontal executive function and cognitive processing speed but they also are sensitive neuropsychological markers associated with worse clinical outcomes related with Alzheimer’s pathologic changes [26]. Notably, general cognitive functions represented by MMSE scores were related with baseline APOE4 allele status and inferior temporal volumes. We can assume that neurodegeneration outside the medial temporal lobes might affect changes in general cognitive functions and may be related with clinical progression to MCI/dementia; although we could observe only small number of progressed participants.

Regarding cognitive progress according to the plasma amyloid beta values, the cognitive trajectories did not differ between plasma Aβ- and plasma Aβ + participants. Although plasma amyloid values were significantly higher in the Aβ + SCD participants and blood-based biomarkers are expected to be potential screening and monitoring markers, plasma amyloid beta values may not predict cognitive declines during relatively short-term follow-up in cognitively unimpaired subjects.

Framingham cardiovascular risk score was developed to predict 10-year risk of cardiovascular events, which includes age, systolic blood pressure, cholesterol, blood pressure medication, smoking, and diabetes. Previous studies have reported that more cardiovascular risk factors are associated with poorer cognitive outcomes and risks of AD through numerous mechanisms [27, 28]. The risk factors may change brain structures and induce small vessel disease. In our study participants, cardiovascular risk scores were higher in Aβ + SCD compared with those in Aβ-SCD, consistent with previous studies.

This study had several limitations. First, the follow-up duration was relatively short considering that annual progression rate of SCD is below 10% based on previous cohort study results [8]. Consistent with previous studies, only 9 participants progressed to MCI/dementia. Further studies with longer follow-up durations might help the generalization of our results. Second, other biomarkers assessing combined tau pathologies such as tau PET or plasma/CSF phosphorylated tau values, lifestyle factors associated with clinical progression, and combined other biomarkers associated with neurodegenerative pathologies including α-synuclein or tar DNA binding protein (TDP-43) were not studied. SCD is a heterogeneous condition with multiple pathologies, mood disorders, and personality factors, and thus future studies should include more pathologic biomarkers and considerations of lifestyle factors. Third, the study participants were recruited based on hospital-based settings; hence, our results might show outcomes in “SCD with worry about the cognitive decline,” which may be different from outcomes in SCD of general population.

Despite these limitations, our study also has several strengths. Participants were consecutively recruited in multiple centers in South Korea using a comprehensive neuropsychological test battery and underwent regular biomarker evaluations, allowing us to clarify cognitive and biomarker trajectories of SCD. Our findings suggest that SCD participants with amyloidosis has greater cognitive declines and neurodegenerative changes despite of the relatively short follow-up durations; moreover, neurodegeneration combined with sufficient amyloid burden would influence faster cognitive progression in SCD.

### Supplementary Information

**Additional file 1: Supplementary Table 1.** Baseline characteristics between follow-up completers and dropped-out subjects. **Supplementary Table 2.** Baseline and endpoint amyloid and neurodegenerative biomarker status. **Supplementary Table 3.** Baseline characteristics according to preclinical stages. **Supplementary Table 4.** Cognitive and biomarker status after 24 months according to baseline preclinical stages. **Supplementary Table 5-1.** Relevant baseline factors related with verbal memory delayed recall scores after 24 months. **Supplementary Table 5-2.** Relevant baseline factors related with frontal executive function scores after 24 months. **Supplementary Table 5-3.** Relevant baseline factors related with general cognitive function (MMSE score) after 24 months.

## Data Availability

The datasets used and/or analyzed during the current study are available from the corresponding author on reasonable request.
