# Supplementary material for: Impacts of baseline biomarkers on cognitive trajectories in subjective cognitive decline: the CoSCo prospective cohort study
Source: Alzheimers Res Ther. 2023 Aug 7;15:132. doi: 10.1186/s13195-023-01273-y (PMC10405399; doi:10.1186/s13195-023-01273-y)
Supplement: Supplementary file 1 — Additional file 1: Supplementary Table 1. Baseline characteristics between follow-up completers and dropped-out subjects. Supplementary Table 2. Baseline and endpoint amyloid and neurodegenerative biomarker status. Supplementary Table 3. Baseline characteristics according to preclinical stages. Supplementary Table 4. Cognitive and biomarker status after 24 months according to baseline preclinical stages. Supplementary Table 5-1. Relevant baseline factors related with verbal memory delayed recall scores after 24 months. Supplementary Table 5-2. Relevant baseline factors related with frontal executive function scores after 24 months. Supplementary Table 5-3. Relevant baseline factors related with general cognitive function (MMSE score) after 24 months. [file 13195_2023_1273_MOESM1_ESM.docx]

**Supplementary Table 1.** Baseline characteristics between follow-up completers and dropped-out subjects.

| Variables | Dropped-out | Follow-up completers | *p* |
| --- | --- | --- | --- |
|  | (N=13) | (N=107) |  |
| Female (n,%) | 8 (61.5%) | 59 (55.1%) | 0.886 |
| Age, yr | 72.2 ± 5.8 | 70.7 ± 6.2 | 0.434 |
| Education, yr | 8.2 ± 3.2 | 11.5 ± 4.0 | 0.005* |
| APOE4 existence (n,%) | 4 (30.8%) | 21 (19.6%) | 0.467 |
| K-MMSE score | 27.0 ± 2.2 | 27.3 ± 1.9 | 0.629 |
| DST-Forward_percentile | 73.6 ± 18.8 | 62.6 ± 29.8 | 0.194 |
| K-BNT_percentile | 56.6 ± 32.0 | 60.6 ± 26.5 | 0.616 |
| RCFT_Copy_percentile | 58.5 ± 23.2 | 58.5 ± 21.6 | 0.998 |
| SVLT delayed recall_percentile | 24.8 ± 11.9 | 28.1 ± 14.5 | 0.424 |
| RCFT delayed recall_percentile | 50.9 ± 22.6 | 47.9 ± 24.5 | 0.674 |
| Digit symbol constitution_score | 65.9 ± 27.7 | 60.9 ± 27.0 | 0.529 |
| COWAT phonemic_percentile | 50.5 ± 27.4 | 53.7 ± 28.4 | 0.704 |
| K.TMT-B_percentile | 63.7 ± 15.7 | 61.7 ± 21.8 | 0.742 |
| K-stroop_color reading_percentile | 47.2 ± 32.9 | 54.8 ± 25.6 | 0.330 |
| Mean_hippocampal atrophy (Schelten’s visual rating) | 1.5 ± 0.8 | 1.1 ± 0.9 | 0.134 |
| Framingham.risk score | 11.7 ± 11.6 | 9.1 ± 7.5 | 0.443 |
| Plasma MDS-Amyloid ꞵ | 0.9 ± 0.2 | 1.0 ± 0.2 | 0.152 |
| Global SUVR | 1.1 ± 0.2 | 1.3 ± 0.2 | 0.007* |
| K.ECOG_memory total | 13.5 ± 4.2 | 17.8 ± 5.5 | 0.008* |
| K-ECOG total | 56.5 ± 15.7 | 72.1 ± 21.7 | 0.014* |
| Body fat (%) | 30.0 ± 6.3 | 29.1 ± 8.0 | 0.696 |
| Body muscle (%) | 21.9 ± 4.7 | 24.5 ± 6.4 | 0.157 |
| Visceral fat (%) | 10.3 ± 4.0 | 9.0 ± 3.7 | 0.233 |

*Abbreviations:* APOE4, apolipoprotein epsilon 4; K-MMSE, Korean version of Mini-Mental State Examination; DST, digit span test; BNT, Boston naming test; RCFT, Rey complex figure test; SVLT, Seoul verbal learning test; COWAT, Controlled Oral Word Association Test; TMT, trail making test; MDS, multimer detection system; SUVR, standardized uptake value ratio; ECOG, Everyday cognition

**Supplementary Table 2.** Baseline and endpoint amyloid and neurodegenerative biomarker status.

| Variables | Aβ-SCD | Aβ+SCD | *p* |
| --- | --- | --- | --- |
|  | (N=87) | (N=20) |  |
| ***Baseline regional volumes (Z scores)*** |  |  |  |
| lh_entorhinal | 1.1 ± 1.1 | 0.6 ± 1.4 | 0.079 |
| lh_inferior_temporal | 0.6 ± 1.1 | 0.5 ± 1.0 | 0.724 |
| rh_entorhinal | -0.0 ± 1.0 | -0.5 ± 1.0 | 0.077 |
| rh_inferior_temporal | 0.8 ± 1.1 | 0.9 ± 1.0 | 0.740 |
| lh_hippocampus | 0.1 ± 1.2 | -0.5 ± 1.3 | 0.050 |
| rh_hippocampus | -0.2 ± 1.3 | -0.8 ± 1.2 | 0.070 |
| cingulate | 1.8 ± 1.0 | 1.9 ± 0.7 | 0.548 |
| frontal_lobe | 1.4 ± 1.1 | 2.0 ± 0.7 | 0.008* |
| insula | 0.9 ± 1.2 | 1.0 ± 1.2 | 0.693 |
| occipital_lobe | 1.1 ± 1.2 | 1.0 ± 1.1 | 0.712 |
| parietal_lobe | 1.8 ± 1.0 | 2.0 ± 0.7 | 0.360 |
| temporal_lobe | 1.5 ± 1.1 | 1.7 ± 0.9 | 0.549 |
| amygdala | 0.0 ± 1.1 | -0.2 ± 1.1 | 0.402 |
| caudate | 0.7 ± 1.1 | 0.7 ± 0.9 | 0.848 |
| hippocampus | -0.0 ± 1.3 | -0.6 ± 1.4 | 0.071 |
| pallidum | 0.3 ± 1.2 | -0.4 ± 0.9 | 0.020* |
| putamen | 0.9 ± 1.2 | 0.9 ± 1.0 | 0.953 |
| thalamus | 0.3 ± 1.3 | 0.3 ± 1.1 | 0.952 |
| ***Endpoint regional volumes (Z scores)*** |  |  |  |
| lh_entorhinal | 1.0 ± 1.2 | 0.4 ± 1.5 | 0.039* |
| lh_inferior_temporal | 0.5 ± 1.2 | 0.3 ± 1.1 | 0.449 |
| rh_entorhinal | 0.0 ± 1.0 | -0.6 ± 1.2 | 0.036* |
| rh_inferior_temporal | 0.7 ± 1.2 | 0.6 ± 1.0 | 0.927 |
| lh_hippocampus | 0.0 ± 1.4 | -0.9 ± 1.1 | 0.009* |
| rh_hippocampus | -0.2 ± 1.4 | -1.2 ± 1.3 | 0.007* |
| cingulate | 1.8 ± 1.1 | 1.9 ± 0.7 | 0.598 |
| frontal_lobe | 1.5 ± 1.2 | 2.0 ± 0.7 | 0.051 |
| insula | 0.9 ± 1.2 | 0.9 ± 1.2 | 0.951 |
| occipital_lobe | 1.2 ± 1.2 | 0.8 ± 1.1 | 0.237 |
| parietal_lobe | 1.8 ± 1.2 | 1.9 ± 0.8 | 0.528 |
| temporal_lobe | 1.5 ± 1.2 | 1.4 ± 1.1 | 0.836 |
| amygdala | 0.1 ± 1.2 | -0.4 ± 1.1 | 0.102 |
| caudate | 0.7 ± 1.2 | 0.7 ± 0.9 | 0.949 |
| hippocampus | -0.0 ± 1.4 | -1.0 ± 1.2 | 0.008* |
| pallidum | 0.3 ± 1.3 | -0.5 ± 0.9 | 0.018* |
| putamen | 0.9 ± 1.2 | 0.9 ± 1.0 | 0.861 |
| thalamus | 0.4 ± 1.3 | 0.2 ± 1.2 | 0.586 |
| ***Baseline & endpoint plasma amyloid*** | | | |
| Baseline_Plasma amyloid βeta | 0.851 ± 0.199 | 0.961 ± 0.122 | 0.030* |
| Endpoint_ Plasma amyloid βeta | 0.911 ± 0.189 | 1.024 ± 0.106 | 0.001* |

Data are shown in standardized Z scores adjusted for age, sex, and the individual’s intracranial volume

**Supplementary Table 3.** Baseline characteristics according to preclinical stages.

| Variables | Stage 0 (N=49) | Stage 1 (N=8) | Stage 2 (N=12) | SNAP (N=38) | *p* |
| --- | --- | --- | --- | --- | --- |
| Female (n, %) | 28 (57.14%) | 4 (50.00%) | 3 (25.00%) | 24 (63.16%) | 0.135 |
| Age, yrs | 68.57 ± 4.94 | 72.00 ± 4.96 | 76.92 ± 5.50 | 71.32 ± 6.68 | 0.009* |
| Education, yrs | 10.96 ± 4.06 | 12.50 ± 2.73 | 13.67 ± 3.89 | 11.42 ± 4.08 | 0.430 |
| APOE4 carrier (n, %) | 7 (14.29%) | 5 (62.50%) | 5 (41.67%) | 4 (10.53%) | 0.001* |
| Waist circum, cm | 87.93 ± 10.23 | 79.78 ± 10.60 | 82.77 ± 8.32 | 86.12 ± 7.38 | 0.331 |
| Body fat (%) | 30.57 ± 6.77 | 25.10 ± 8.01 | 26.09 ± 9.39 | 28.87 ± 8.81 | 0.277 |
| Body muscle (%) | 25.17 ± 5.71 | 23.59 ± 5.15 | 23.83 ± 4.76 | 24.05 ± 7.79 | 0.405 |
| Visceral fat (%) | 9.69 ± 4.00 | 6.62 ± 2.83 | 6.50 ± 3.29 | 9.37 ± 3.17 | 0.499 |
| Global SUVR | 1.19 ± 0.11 | 1.58 ± 0.15 | 1.68 ± 0.23 | 1.21 ± 0.11 | 0.240 |
| MDS_Plasma amyloid | 0.83 ± 0.20 | 0.86 ± 0.28 | 1.03 ± 0.13 | 0.88 ± 0.20 | 0.106 |
| Deep WMH (mild/moderate/severe) | 39/8/2 | 7/1/0 | 9/3/0 | 25/8/5 | 0.455 |
| Periventricle WMH (mild/moderate/severe) | 37/9/3 | 5/2/1 | 8/1/3 | 23/8/7 | 0.559 |
| Lacunes (n) | 0.06±0.24 | 0.00±0.00 | 0.08±0.29 | 0.13±0.41 | 0.635 |
| Microbleed (n) | 0.06±0.24 | 0.13±0.35 | 0.17±0.39 | 0.05±0.23 | 0.551 |
| Framingham cardiovascular risk score | 8.69 ± 7.30 | 7.84 ± 8.07 | 15.89 ± 7.93 | 7.73 ± 6.49 | 0.998 |
| Baseline K-MMSE score | 27.49 ± 1.80 | 27.88 ± 1.25 | 26.33 ± 2.42 | 27.18 ± 2.04 | 0.294 |
| DST-Forward_percentile | 59.25 ± 29.00 | 68.14 ± 29.73 | 54.84 ± 34.09 | 68.12 ± 29.47 | 0.244 |
| K-BNT_percentile | 64.40 ± 24.96 | 66.99 ± 31.63 | 56.27 ± 34.40 | 55.70 ± 24.56 | 0.102 |
| RCFT Copy_percentile | 58.08 ± 22.64 | 70.04 ± 18.07 | 50.75 ± 21.85 | 59.09 ± 20.51 | 0.927 |
| SVLT delayed recall_percentile | 29.39 ± 15.02 | 22.41 ± 15.20 | 18.54 ± 10.83 | 30.67 ± 13.55 | 0.933 |
| RCFT delayed recall_percentile | 53.50 ± 24.81 | 42.58 ± 29.76 | 46.74 ± 23.07 | 42.14 ± 22.75 | 0.035* |
| Digit symbol constitution_score | 68.59 ± 24.88 | 63.40 ± 30.62 | 41.57 ± 34.49 | 56.59 ± 23.22 | 0.013* |
| COWAT phonemic_percentile | 58.92 ± 27.58 | 45.41 ± 32.43 | 61.80 ± 29.54 | 46.13 ± 27.18 | 0.067 |
| K-TMT-B_percentile | 62.84 ± 21.17 | 68.50 ± 13.05 | 57.26 ± 23.69 | 60.08 ± 23.75 | 0.456 |
| K-stroop color reading_percentile | 55.85 ±25.87 | 67.41 ± 23.85 | 37.67 ± 28.10 | 56.09 ± 23.25 | 0.666 |
| K-ECOG memory total | 17.49 ±5.93 | 20.25 ± 3.73 | 16.58 ± 5.09 | 18.05 ± 5.41 | 0.798 |
| K-ECOG total | 69.12 ± 21.14 | 75.62 ± 21.67 | 69.75 ± 20.37 | 75.92 ± 22.96 | 0.183 |

*Abbreviations:* SCD, subjective cognitive decline; APOE4, apolipoprotein epsilon 4; SUVR, standardized uptake value ratio; MDS, multimer detection system; WMH, white matter hyperintensities; K-MMSE, Korean version of Mini-Mental State Examination; DST, digit span test; BNT, Boston naming test; RCFT, Rey complex figure test; SVLT, Seoul verbal learning test; COWAT, Controlled Oral Word Association Test; TMT, trail making test; ECOG, Everyday cognition.

**Supplementary Table 4.** Cognitive and biomarker status after 24 months according to baseline preclinical stages.

| Variables | Stage 0 (N=49) | Stage 1 (N=8) | Stage 2 (N=12) | SNAP (N=38) | *p* |
| --- | --- | --- | --- | --- | --- |
| ***Cognitive scores at 24 months*** | | | | | |
| DST-Forward_percentile | 64.93 ± 29.37 | 74.76 ± 21.51 | 54.55 ± 35.39 | 72.63 ± 26.93 | 0.367 |
| K-BNT_percentile | 71.70 ± 23.65 | 66.32 ± 41.48 | 50.32 ± 34.70 | 58.03 ± 30.27 | 0.016* |
| RCFT Copy_percentile | 54.45 ± 26.41 | 57.32 ± 20.01 | 54.11 ± 26.14 | 49.06 ± 24.16 | 0.329 |
| SVLT delayed recall_percentile | 49.18 ± 26.26 | 34.64 ± 24.99 | 29.81 ± 24.12 | 53.36 ± 25.50 | 0.681 |
| RCFT delayed recall_percentile | 60.92 ± 29.56 | 59.24 ± 32.43 | 48.33 ± 26.42 | 55.02 ± 28.66 | 0.265 |
| Digit symbol constitution_score | 70.73 ± 26.40 | 80.99 ± 26.15 | 48.98 ± 33.89 | 65.47 ± 23.66 | 0.172 |
| COWAT phonemic_percentile | 55.03 ± 30.51 | 65.07 ± 30.57 | 56.28 ± 30.94 | 55.36 ± 28.34 | 0.998 |
| K-TMT-B_percentile | 67.71 ± 19.30 | 52.38 ± 26.87 | 41.02 ± 26.87 | 67.12 ± 23.46 | 0.561 |
| K-stroop color reading_percentile | 60.88 ± 29.99 | 67.27 ± 16.16 | 43.54 ± 29.77 | 60.07 ± 27.29 | 0.619 |
| K-MMSE score | 28.08 ± 1.69 | 28.12 ± 1.81 | 25.75 ± 3.05 | 28.00 ± 1.90 | 0.437 |
| ***Biomarker status at 24 months*** | | | | | |
| MDS_Plasma amyloid βeta | 0.88 ± 0.18 | 0.99 ± 0.10 | 1.04 ± 0.11 | 0.95 ± 0.20 | 0.002* |
| Left entorhinal vol (z score) | 1.54 ± 0.95 | 1.16 ± 1.24 | -0.17 ± 1.38 | 0.39 ± 1.21 | <0.001* |
| Left inferior temporal vol (z score) | 0.85 ± 1.03 | 0.94 ± 1.00 | -0.14 ± 0.94 | 0.13 ± 1.38 | 0.003* |
| Right entorhinal vol (z score) | 0.53 ± 0.95 | 0.25 ± 1.01 | -1.09 ± 0.96 | -0.61 ± 0.79 | <0.001* |
| Right inferior temporal vol (z score) | 1.06 ± 1.04 | 1.02 ± 1.14 | 0.40 ± 0.93 | 0.20 ± 1.25 | <0.001* |
| Left hippocampal vol (z score) | 0.78 ± 0.90 | 0.03 ± 0.78 | -1.45 ± 0.93 | -0.90 ± 1.27 | <0.001* |
| Right hippocampal vol (z score) | 0.56 ± 1.04 | -0.40 ± 1.17 | -1.69 ± 1.08 | -1.16 ± 1.20 | <0.001* |
| Cingulate vol (z score) | 2.04 ± 0.58 | 2.18 ± 0.41 | 1.71 ± 0.82 | 1.49 ± 1.47 | 0.091 |
| Frontal lobe vol (z score) | 1.87 ± 0.75 | 2.18 ± 0.42 | 1.82 ± 0.88 | 1.11 ± 1.57 | 0.026* |
| Insula vol (z score) | 1.31 ± 0.90 | 1.74 ± 0.83 | 0.36 ± 1.14 | 0.47 ± 1.44 | 0.004* |
| Occipital lobe vol (z score) | 1.62 ± 0.63 | 1.19 ± 0.93 | 0.61 ± 1.12 | 0.68 ± 1.54 | 0.009* |
| Parietal lobe vol (z score) | 2.07 ± 0.60 | 2.20 ± 0.37 | 1.77 ± 0.95 | 1.40 ± 1.53 | 0.085 |
| Temporal lobe vol (z score) | 1.87 ± 0.79 | 2.11 ± 0.61 | 0.99 ± 1.13 | 1.05 ± 1.49 | 0.001* |
| Amygdala vol (z score) | 0.67 ± 0.89 | 0.43 ± 0.84 | -0.98 ± 0.91 | -0.65 ± 1.16 | <0.001* |
| Caudate vol (z score) | 0.89 ± 1.02 | 1.02 ± 0.54 | 0.45 ± 0.99 | 0.37 ± 1.31 | 0.027* |
| Pallidum vol (z score) | 0.49 ± 1.12 | -0.10 ± 1.11 | -0.72 ± 0.65 | -0.03 ± 1.38 | 0.027* |
| Putamen vol (z score) | 1.16 ± 0.92 | 1.12 ± 0.90 | 0.73 ± 1.03 | 0.66 ± 1.43 | 0.415 |
| Thalamus vol (z score) | 0.57 ± 1.22 | 0.81 ± 1.25 | -0.24 ± 0.96 | 0.10 ± 1.46 | 0.061 |

*Abbreviations:* MDS, multimer detection system; K-MMSE, Korean version of Mini-Mental State Examination; DST, digit span test; BNT, Boston naming test; RCFT, Rey complex figure test; SVLT, Seoul verbal learning test; COWAT, Controlled Oral Word Association Test; TMT, trail making test

**Supplementary Table 5-1.** Relevant baseline factors related with verbal memory delayed recall scores after 24 months.

| Variables | Univariable | | | Multivariable | | |
| --- | --- | --- | --- | --- | --- | --- |
|  | ß | 95% CI | *P* | ß | 95% CI | *P* |
| Global SUVR, ratio | -0.242 | -52.071~-6.578 | 0.012 |  |  |  |
| Age, yr | -0.061 | -1.092~0.566 | 0.530 |  |  |  |
| Female | 0.139 | -2.811~17.517 | 0.154 |  |  |  |
| Education, yr | -0.065 | -1.707~0.843 | 0.503 |  |  |  |
| Baseline body muscle | -0.025 | -0.910~0.702 | 0.799 |  |  |  |
| Baseline body fat | 0.046 | -0.487~0.789 | 0.640 |  |  |  |
| Baseline SVLT delayed recall (percentile) | 0.499 | 0.607~1.221 | <0.001 | 0.462 | 0.535~1.158 | <0.001 |
| K-ECOG memory score | -0.087 | -1.345~0.507 | 0.372 |  |  |  |
| MDS plasma amyloid | -0.061 | -32.818~17.182 | 0.536 |  |  |  |
| APOE4 | -0.149 | -22.621~2.793 | 0.125 |  |  |  |
| Microbleed, n | 0.049 | -14.449~24.317 | 0.615 |  |  |  |
| Lacune, n | 0.042 | -12.872~20.093 | 0.665 |  |  |  |
| WMH, periventricular | 0.084 | -3.941~10.075 | 0.387 |  |  |  |
| WMH, deep | 0.100 | -4.151~13.076 | 0.307 |  |  |  |
| left entorhinal vol. | 0.250 | 4.492~31.698 | 0.010 | 0.166 | -0.286~24.267 | 0.055 |
| Hippocampal vol. | 0.087 | -5.934~15.518 | 0.378 |  |  |  |

*Abbreviations:* APOE4, apolipoprotein epsilon 4; SUVR, standardized uptake value ratio; MDS, multimer detection system; WMH, white matter hyperintensities; SVLT, Seoul verbal learning test; ECOG, Everyday cognition

**Supplementary Table 5-2.** Relevant baseline factors related with frontal executive function scores after 24 months.

| Variables | Univariable | | | Multivariable | | |
| --- | --- | --- | --- | --- | --- | --- |
|  | ß | 95% CI | *P* | ß | 95% CI | *P* |
| Global SUVR, ratio | -0.379 | -60.553~ -21.722 | <0.001 | -0.410 | -61.653~ -27.504 | <0.001 |
| Age, yr | -0.248 | -1.672~ -0.231 | 0.010 |  |  |  |
| Female | 0.013 | -8.571~ 9.800 | 0.895 |  |  |  |
| Education, yr | 0.082 | -0.654~ 1.626 | 0.400 |  |  |  |
| Baseline body muscle | 0.171 | -0.074~ 1.348 | 0.079 |  |  |  |
| Baseline body fat | -0.034 | -0.673~ 0.470 | 0.726 |  |  |  |
| Baseline MMSE score | 0.209 | 0.246~ 4.858 | 0.030 |  |  |  |
| Baseline K-TMT-B (%) | 0.420 | 0.266~ 0.648 | <0.001 | 0.396 | 0.261~ 0.602 | <0.001 |
| K-ECOG executive score | 0.002 | -0.521~ 0.533 | 0.983 |  |  |  |
| MDS plasma amyloid | 0.026 | -19.639~ 25.666 | 0.792 |  |  |  |
| APOE4 | -0.094 | -17.065~ 5.839 | 0.333 |  |  |  |
| Microbleed, n | -0.040 | -20.948~ 13.766 | 0.682 |  |  |  |
| Lacune, n | 0.017 | -13.432~ 16.097 | 0.858 |  |  |  |
| WMH, periventricular | -0.107 | -9.732~ 2.788 | 0.274 |  |  |  |
| WMH, deep | 0.055 | -5.547~ 9.928 | 0.576 |  |  |  |
| Baseline frontal vol. | 0.218 | 0.040~ 0.578 | 0.025 | 0.237 | 0.112~ 0.558 | 0.004 |
| Baseline entorhinal vol | 0.140 | -3.404~ 21.659 | 0.152 |  |  |  |

*Abbreviations:* APOE4, apolipoprotein epsilon 4; SUVR, standardized uptake value ratio; MMSE, Mini-Mental State Examination; MDS, multimer detection system; WMH, white matter hyperintensities; ECOG, Everyday cognition.

**Supplementary Table 5-3.** Relevant baseline factors related with general cognitive function (MMSE score) after 24 months.

| Variables | Univariable | | | Multivariable | | |
| --- | --- | --- | --- | --- | --- | --- |
|  | ß | 95% CI | *P* | ß | 95% CI | *P* |
| Global SUVR, ratio | -0.245 | -4.082~ -0.532 | 0.011 |  |  |  |
| Female | -0.082 | -1.143~ 0.460 | 0.401 |  |  |  |
| Baseline body muscle | 0.183 | -0.003~ 0.121 | 0.061 |  |  |  |
| Baseline body fat | -0.069 | -0.067~ 0.032 | 0.483 |  |  |  |
| Baseline MMSE score | 0.376 | 0.208~ 0.592 | <0.001 | 0.343 | 0.188~ 0.544 | <0.001 |
| K-ECOG total score | 0.076 | -0.011~ 0.026 | 0.439 |  |  |  |
| MDS plasma amyloid | -0.152 | -3.512~ 0.431 | 0.124 |  |  |  |
| APOE4 | -0.232 | -2.219~ -0.232 | 0.017 | -0.245 | -2.165~ -0.420 | 0.004 |
| Microbleed, n | 0.063 | -1.018~ 2.008 | 0.518 |  |  |  |
| Lacune, n | -0.002 | -1.301~ 1.276 | 0.984 |  |  |  |
| WMH, periventricular | -0.171 | -1.027~ 0.057 | 0.079 |  |  |  |
| WMH, deep | 0.024 | -0.594~ 0.759 | 0.810 |  |  |  |
| Baseline left entorhinal vol. | 0.318 | 0.750~ 2.852 | 0.001 | 0.167 | -0.063~ 1.956 | 0.066 |
| Baseline left inferior temporal vol. | 0.301 | 0.172~ 0.733 | 0.002 | 0.239 | 0.096~ 0.625 | 0.008 |
| Baseline frontal vol | 0.213 | 0.003~ 0.050 | 0.029 |  |  |  |

*Abbreviations:* APOE4, apolipoprotein epsilon 4; SUVR, standardized uptake value ratio; MMSE, Mini-Mental State Examination; MDS, multimer detection system; WMH, white matter hyperintensities; ECOG, Everyday cognition.
